# HA-g-CS Implant and Moderate-intensity Exercise Stimulate Subchondral Bone Remodeling and Promote Repair of Osteochondral Defects in Mice

**DOI:** 10.7150/ijms.63401

**Published:** 2021-10-22

**Authors:** Ke Shen, Xiaonan Liu, Hanjun Qin, Yu Chai, Lei Wang, Bin Yu

**Affiliations:** 1 Department of Orthopaedics, Nanfang Hospital, Southern Medical University, Guangzhou, Guangdong 510515, China.; 2 Key Laboratory of Bone and Cartilage Regeneration Medicine, Southern Medical University, Guangzhou, Guangdong 510515, China.

## Abstract

**Background:** Substantial evidence shows that crosstalk between cartilage and subchondral bone may play an important role in cartilage repair. Animal models have shown that hydroxyapatite-grafted-chitosan implant (HA-g-CS) and moderate-intensity exercise promote regeneration of osteochondral defects. However, no* in vivo* studies have demonstrated that these two factors may have a synergistic activity to facilitate subchondral bone remodeling in mice, thus supporting bone-cartilage repair.

**Questions:** This study was to clarify whether HA-g-CS and moderate-intensity exercise might have a synergistic effect on facilitating (1) regeneration of osteochondral defects and (2) subchondral bone remodeling in a mouse model of osteochondral defects.

**Methods:** Mouse models of osteochondral defects were created and divided into four groups. BC Group was subjected to no treatment, HC Group to HA-g-CS implantation into osteochondral defects, ME group to moderate-intensity treadmill running exercise, and HC+ME group to both HA-g-CS implantation and moderate-intensity exercise until sacrifice. Extent of subchondral bone remodeling at the injury site and subsequent cartilage repair were assessed at 4 weeks after surgery.

**Results:** Compared with BC group, HC, ME and HC+ME groups showed more cartilage repair and thicker articular cartilage layers and HC+ME group acquired the best results. The extent of cartilage repair was correlated positively to bone formation activity at the injured site as verified by microCT and correlation analysis. Histology and immunofluorescence staining confirmed that bone remodeling activity was increased in HC and ME groups, and especially in HC+ME group. This bone formation process was accompanied by an increase in osteogenesis and chondrogenesis factors at the injury site which promoted cartilage repair.

**Conclusions:** In a mouse model of osteochondral repair, HA-g-CS implant and moderate-intensity exercise may have a synergistic effect on improving osteochondral repair potentially through promotion of subchondral bone remodeling and generation of osteogenesis and chondrogenesis factors.

**Clinical Relevance:** Combination of HA-g-CS implantation and moderate-intensity exercise may be considered potentially in clinic to promote osteochondral defect repair. Also, cartilage and subchondral bone forms a functional unit in an articular joint and subchondral bone may regulate cartilage repair by secreting growth factors in its remodeling process. However, a deeper insight into the exact role of HA-g-CS implantation and moderate-intensity exercise in promoting osteochondral repair in other animal models should be explored before they can be applied in clinic in the future.

## Background

Since articular cartilage is avascular tissue with its nutrition provided mainly by synovial fluid, its regeneration is very limited 1. Osteochondral lesions, if untreated for a long-term, are frequently associated with disability and with symptoms manifested by joint pain, locking phenomena and disturbed joint function. Moreover, such lesions may progress to severe forms of osteoarthritis 2, 3. Although much progress has been made in repair of osteochondral defects, treatment of osteocartilage lesions remains complex and challenging. Since cartilage tissue is unlikely to recover by itself, surgical approaches, like implantation of biocompatible grafts to facilitate the healing process, are necessary to repair the damaged cartilage tissue 4. Currently, the application of articular cartilage repair materials has been reported according to different defect sizes. Cartilage defects less than 4cm^2^ in size can be repaired by bone marrow stimulation techniques, including the concomitant injection of biologics (such as growth factors, bone morph protein 4 or 7), the use of acellular scaffoles (such as collagen membranes) or liquid hydrogels, and the use of micropowdered acellular chondrocyte extracellular matrix from allografts^5^. For cartilage repair with a defect of less than 2cm^2^, autografts and allografts achieved a high satisfaction rate of long-term graft survival 6-8. Currently, cell-based cartilage repair techniques are quite attractive for cartilage repair with a defect larger than 3-4cm^2^, especially for matrix induced autologous chondrocyte implantation (MACI), It has been reported that knee patients who underwent MACI surgery experienced significant pain reduction 5 years after surgery^9^, ^10^. Although there are many methods for cartilage repair, the uncertainty of their efficacy and their disadvantages are still obvious. In addition, current grafts for osteochondral repair are limited in availability and often fail due to insufficient integration into the host 1, 11, 12. Also, these engineered grafting substitutes can hardly promote host tissue regeneration and remodeling 13. Therefore, we hope to provide a new idea of cartilage repair by combining materials with functional repair of patients' own cells.

The hydrogel systems have been reported to be effective in promoting articular cartilage 14, 15 and bone regeneration as well 16 because of their physiochemical similarity to native extracellular matrix which is beneficial for retaining native environment for cells 17. Also, the hydrogel systems provide a drug delivery platform as they can incorporate bioactive molecules and protect them from rapid degradation *in vivo*, facilitating tissue regeneration in a long run 18. More recently, chitosan hydrogels have been developed as cartilage tissue engineering scaffolds 19, 20 because the structure of chitosan is similar to glycosaminoglycans (GAGs), one of the main components in normal articular cartilage 21, 22, thus demonstrating superb biocompatibility 23. Hydroxyapatite is the major mineral component in animal bone tissue that has both osteoconductive and osteoinductive properties 24. Studies have shown that addition of hydroxyapatite to hydrogel can increase liquid resorption activity of a graft, thus facilitating cell and nutrient infiltration 25. In our previous studies, to enhance mechanical stability and bioactivity of a graft, we used hydroxyapatite-grafted-chitosan hydrogel to promote tissue-specific interactions between the graft and the injured tissue 23, 26.

Exercise intensity may be another factor affecting cartilage repair. Previous studies have found that moderate-intensity exercise can help articular cartilage recover from injury while high-intensity exercise, on the contrary, may lead to cartilage degeneration 3, 26-28. A previous study also supported this notion that moderate-intensity exercise after injury can significantly promote healing of cartilage defects while delayed intervention by moderate exercise may reduce its benefits in repairing the defects 27. Some researchers have demonstrated that exercise training can mobilize mesenchymal cells from subchondral bone and produce more regenerative tissue 29. On the contrary, 6 days of immobilization resulted in a 41% reduction in proteoglycan synthesis and when the immobilization continued for 3 weeks there was a total loss of proteoglycan aggregates 30. Thus, in this study, we tried to combine treatment of HA-g-CS grafts with moderate-intensity treadmill exercise to see if they may have a synthetic effect on repair of full-thickness osteochondral defects in mouse models of the knee joint.

When exploring the intrinsic factors affecting the regeneration process, researchers have demonstrated the importance of subchondral bone during the repair of full thickness chondral defects 31, 32. Normally, subchondral bone provides both osseous and nutrient supports for cartilage 34. In articular osteochondral models involving microfractures, remodeling of upward subchondral bone plate is commonly reported 32. Also, structural changes in subchondral bone, such as osteoarthritis 34, can significantly alter chondrocytes behavior 35. Thus, cartilage and its underling subchondral bone should be considered together to ensure a successful cartilage repair 36. However, it is unknown whether HA-g-CS implantation or moderate-intensity exercise may affect the interaction between subchondral bone at the injury site and the repaired cartilage.

The present study sought to evaluate the effect of HA-g-CS implantation and/or moderate-intensity exercise in osteochondral defect regeneration. The subchondral bone change and its relationship with the extent of cartilage repair were also assessed. We quantified the growth factors released in subchondral bone and repaired cartilage tissue, respectively, and evaluated the overall effect of the treatments on joint morphology and pain response.

## Material and Methods

### Animals and treatment

We purchased 3-months-old C57/BL6 mice strain from Model Animal Research Center at Southern Medical University (Guangzhou, China). General condition of the mice was carefully evaluated and monitored by veterinary examination. All animals were maintained in the animal facility at Nanfang Hospital, Southern Medical University. The experimental protocol was reviewed and approved by Nanfang Hospital Animal Ethic Committee.

Thirty-two mice were randomly divided into the following four groups (n=8 in each group), BC: blank control group; HC: HA-g-CS implant group; ME: moderate-intensity exercise group; HC+ME: HA-g-CS implant combined with moderate-intensity exercise group. Mice in ME and HC+ME group were subjected to treadmill exercise after a rest for 1 week after surgery. Running intensity was set as moderate according to the maximum of oxygen consumption 27, 37. Concisely, the mice were made running for 45 min on a 11u grade treadmill at a speed of 18.5 m/min with the slope 5°, once per day and 5 days a week for four weeks. All groups of mice were euthanized at the same time and subsequently knee specimens were collected for further experiments.

### Cartilage defect model and HA-g-CS implantation

**Figure 1:**
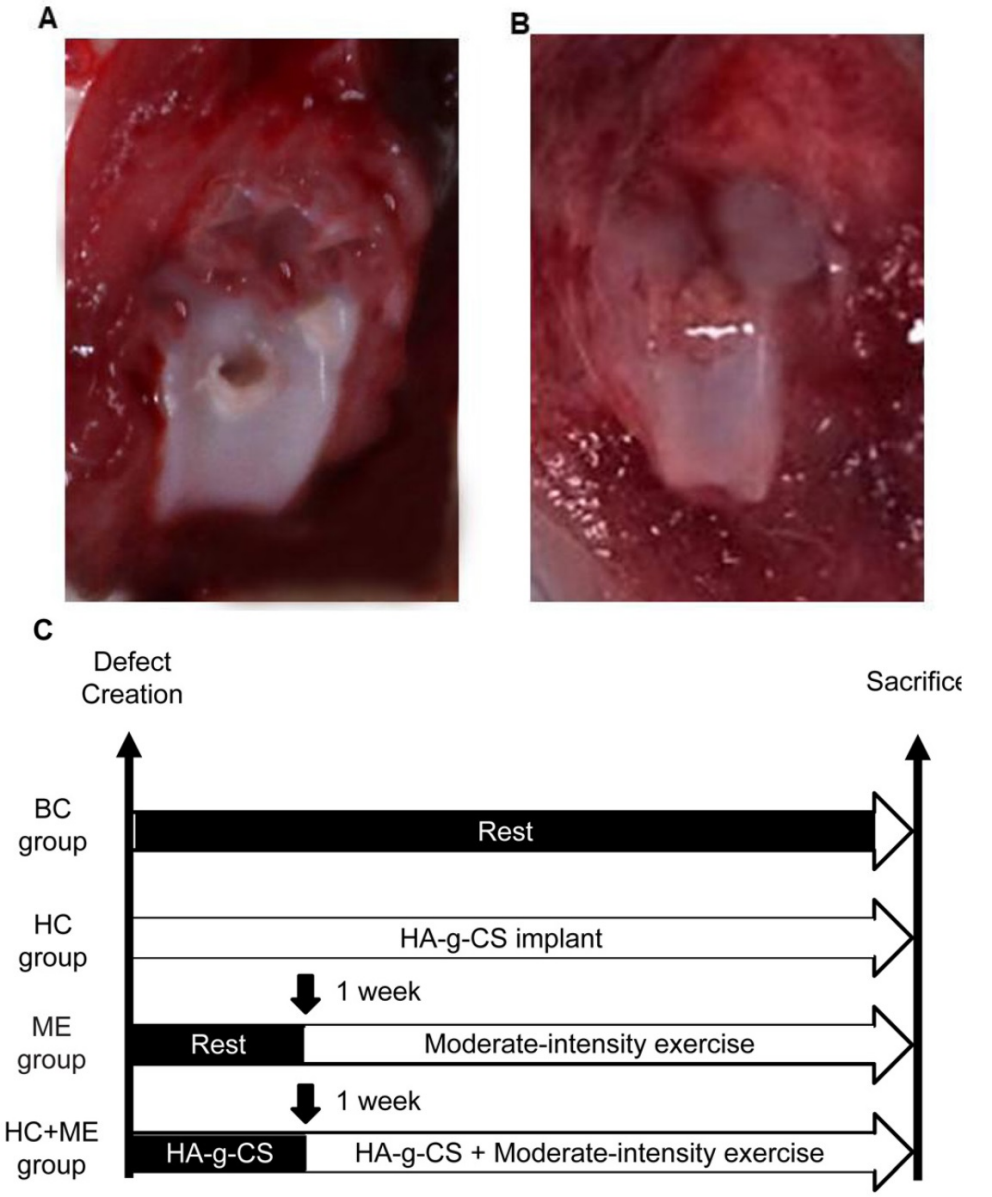
Generation of an osteochondral defect model in mice and study design. A full-thickness articular cartilage defect was generated in the central (weight-bearing) area of the medial femoral condyle in the mouse model (A). Models were treated by HA-g-CS implantation (B), moderate-intensity exercise and combination of the two treatments. Study design of the four groups was shown (C). BC: blank control group; HC: HA-g-CS implantation group; ME: moderate-intensity exercise group; HC+ME: HA-g-CS implantation plus moderate-intensity exercise group.

A full thickness osteochondral defect was created at the knee joint in each mouse using a standard operative procedure as previously described 27, 38. Generally, the mice were put under general anesthesia (sodium pentobarbital 0.2 m1/100 g body weight, i.p.) before a medial parapatellar incision was made to dislocate the patella laterally. The joint was temporarily flexed to expose the femoral trochlea. The articular cartilage at the middle of the femoral trochlea was perforated with a 0.5mm hand drill until bleeding when the drill reached the subchondral bone but did not damage the bone surface (Figure 1A). The joint was washed with a sterile saline solution (0.9% NaCl) to remove the cartilaginous and osseous debris. In HC and HC+ME groups, HA-g-CS generated as preciously described 39 was implanted immediately after surgery (Figures 1B). After the medial capsular incision was carefully closed by suture, the mice were carefully observed for 24h for any sign of infection or bleeding.

### Articular surface observation and histological evaluation

For gross observation of the articular surface, all the soft tissues around the knee joint were removed from the distal femur after sacrifice of the animals. Samples were imaged with a high-resolution camera (Canon, Japan). Macroscopic evaluation of the repaired tissue was conducted and scored with Wayne's grading scale or ICRS score. Each sample was independently evaluated by two investigators.

For staining of frozen sections, after decalcification with 0.5M EDTA for 2 weeks, the bones were immersed in 20% sucrose and 2% polyvinylpyrrolidone solution and dehydrated for 24 hours. After the tissues were embedded in OCT, 20-μm-thick sections were collected for staining. For immunofluorescence staining, the sections were incubated with primary antibodies to Emcn (Santa-Cruz, sc-65495, 1:100), CD31 (R&D Systems, FAB3628G, 1:200), osterix (Abcam, ab22552, 1:100), and osteocalcin (Takara, M188, 1:200) overnight at 4°C, and then with second antibodies. Nuclei were counterstained with DAPI (Vector Laboritories, H-1200, USA) and observed under a Zeiss LSM780 confocal microscope. TRAP staining kit (Sigma, 387A-1KT, USA) was used to stain and calculate TRAP^+^ mature osteoclast cells. For each sample, 5 different fields in both primary spongiosa and secondary spongiosa were calculated.

For Safranin-O & fast green staining, after fixation and decalcification, the samples were embedded in paraffin and sectioned at 4 µm, followed by Safranin-O & fast green staining 40.

### Micro-CT analysis

For micro-CT analysis, the mice femur was fixed overnight in 10% formalin at 4°C and then scanned and analyzed by a high-resolution micro-CT (Bruker MicroCT, Skyscan 1175, Belgium). The X-ray was set at 65 kV, 153 μA, and a resolution of 11.0 μm/pixel. We used NRecon image reconstruction software (version 1.6, Bruker MicroCT) and CTAn data-analysis software (version 1.9, Bruker MicroCT) to reconstruct and analyze the subchondral trabecular bone at the injury site. To select the region of interest (ROI) in analysis of the trabecular bone, we first identified the subchondral bone and drew the regions of interest from below the injured articular surface (with a diameter of 1mm) and extended toward the distal direction for proximally 0.7mm in length. The trabecular bone was analyzed to determine trabecular BV/TV, Tb. Th, Tb. N, and Tb. Sp.

### Quantitative Real-Time PCR

Total RNA for qRT-PCR was extracted from the subchondral bone at the injury site or repaired cartilage tissue using RNeasy Mini Kit (QIAGEN, USA) according to the manufacturer's protocol. In order to ensure the consistency of sampling sites, all PCR sites were sampled within 2mm of the defect edge. cDNA was prepared and analyzed with SYBR GreenMaster Mix (QIAGEN, USA) in the thermal cycler with forward and reverse primers specific for each targeted gene. Target-gene expression was normalized to glyceraldehyde 3-phosphate dehydrogenase (GAPDH) messenger RNA, and relative gene enrichment was assessed using the 2-ΔΔCT method. Primers used for qRT-PCR were as follows: IGF-1 (5′- AAAGCAGCCCCGCTCTATCC-3′) and (5′- CTTCTGAGTCTTGGGCATGTCA-3′); BMP-2 (5′- ATGGATTCGTGGTGGAAGTG-3′) and (5′- GTGGAGTTCAGATGATCAGC-3′); b-FGF (5′- CCGCCCTGCCGGAGGATGGAGGCA-3′) and (5′- GCCTTCTGCCCAGGTCCTGT-3′); SOX-9 (5′- AGTACCCGCATCTGCACAAC -3′) and (5′-TACTTGTAATCGGGGTGGTCT-3′); Collagen II (5′- CAGGTGAACCTGGACGAGAG-3′) and (5′- ACCACGATCTCCCTTGAC TC-3′); N-Cadh (5′-GTGCCATTAGCCAAGGGAATTCAGC-3′) and (5′- GCGTTCCTGTTCCACTCATAGGAGG-3′).

### Chemical analyses of the cartilage

Measurement of DNA content and glycosaminoglycan (GAG) content was done as follows: First, samples were dried at 37°C for 48 hours and then digested in papain solution at 60°C for 24 hours. The total DNA content was examined by the Quit-iT dsDNA kit (Invitrogen, USA). Standard curve was generated using the salmon testes DNA (Sigma, USA). The glycosaminoglycan (GAG) content was examined using the dimethylmethylene blue assay. Standard curve was generated by the chondroitin sulfate from shark cartilage (Sigma, USA).

### Behavior tests

All behavior tests were done at 4 weeks after surgery. For wheel running test, the distance traveled was measured using an activity wheel monitoring system (Lafayette Instruments, USA). Mice were housed singly in a cage that contained an activity wheel which could record the total distance as mice traveled in the wheels. Each mouse had 24 hr voluntary access to its own running wheel during the experiment.

For tactile sensitivity test, mice were acclimated for 30 min in the test chamber on a wire grid platform before the von Frey testing. Mechanical sensitivity was measured by determining the threshold of hind paw withdrawal using a set of 17 von Frey filaments (Lafayette Instruments, USA) with ascending force intensities. The force applied on the monofilament that might elicit pain increased from 0.026 g in the first handle of the set to 110 g in the last. Positive responses were defined as a rapid withdrawal of the hind paw, and the number of positive responses for each stimulus was recorded. Each mouse was assessed three times and relative changes (percentage) from baseline readings were reported.

### Statistics

Data are presented as means ± standard errors of the mean. For multiple comparisons, one-way analysis of variance (ANOVA) with Bonferroni post hoc test was used. All data were normally distributed and had similar variation between groups. Statistical analysis was performed using SAS, version 9.3, software (SAS Institute, NC). *p*< 0.05 was deemed significant.

## Results

### Effects of HA-g-CS implantation and/or moderate-intensity exercise on cartilage repair

**Figure 2:**
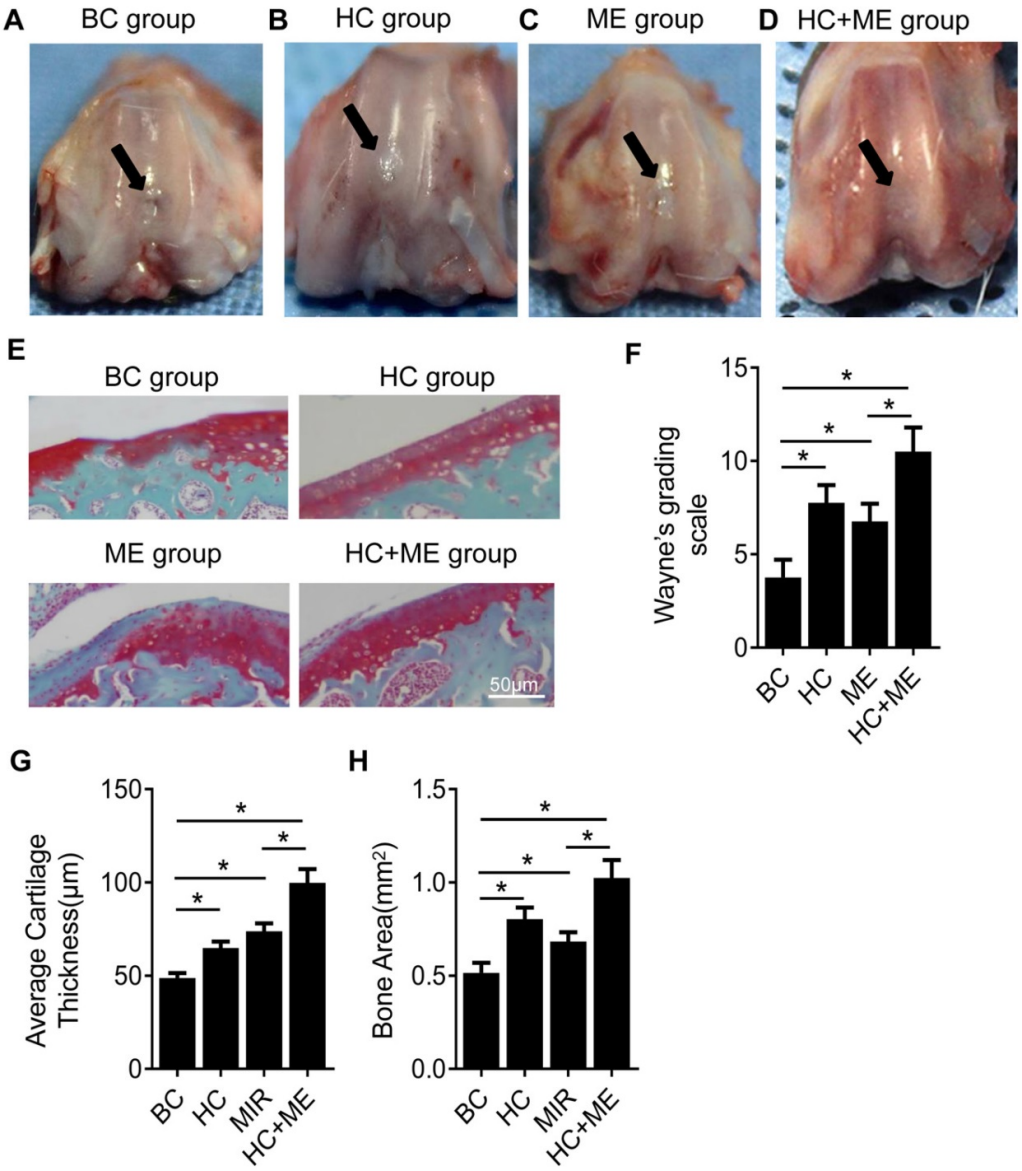
General appearance and Safranin-O & fast green staining showing the effect of HAg-CS implantation and/or moderate-intensity exercise on cartilage repair. The macroscopic appearance showed the repaired cartilage tissue in the healing wound in BC group (A), HC group (B), ME group (C) and HC+ME group (D). Safranin-O & fast green staining of cartilage repair in the four groups was shown (E). The general appearance of articular surface was graded by Wayne's grading scale (F). The articular cartilage thickness (G) and bone area (H) were calculated from the pictures. Data are represented as mean ± s.e.m. (* p<0.05)

We first performed gross observation of the cartilage surface in the four groups. At 4 weeks after surgery, no sign of infection or osteoarthritis was observed in any group. However, repair of the defects on cartilage surface was observed to different degrees in each group. In BC group, moderate joint adhesion was observed (data not shown) but the cartilage defects were not restored, with the subchondral bone still visible (Figure 2A). In HC group, the repaired tissue did not form a smooth articular surface but the joint defect was filled with neo-cartilage tissue and the subchondral bone was not exposed in the central part (Figure 2B). ME group also showed a small amount of repaired cartilage tissue but the central defect was still not completely repaired and a mild collapse was observed in the center of the defect (Figure 2C). HC+ME group showed that the cartilage defect was largely restored in shape with smooth-surfaced tissue, collapse of the defect was shallower compared with the other groups, and the subchondral bone was not exposed (Figure 2D). The extent of cartilage defect repair was quantified by the modified Wayne's grading scale 41 ( Figure 2F).

Safranin-O and fast green staining showed that the cartilage defect was obvious in BC group but partially repaired in HC and ME groups and almost totally restored in HC+ME group (Figure 2E). The average articular cartilage thickness was calculated from the staining pictures in figure 2G. Interestingly, HA-g-CS implantation and/or moderate-intensity exercise also increased bone formation activity in the subchondral bone at the injury site as HC, ME and HC+ME groups had more bone matrix as shown in the blue color in safranin-O & fast green staining (Figure 2E) and calculated bone area in figure 2H.

### HA-g-CS implantation and/or moderate-intensity exercise stimulate bone formation in the subchondral bone at the injury site in the mouse model

**Figure 3:**
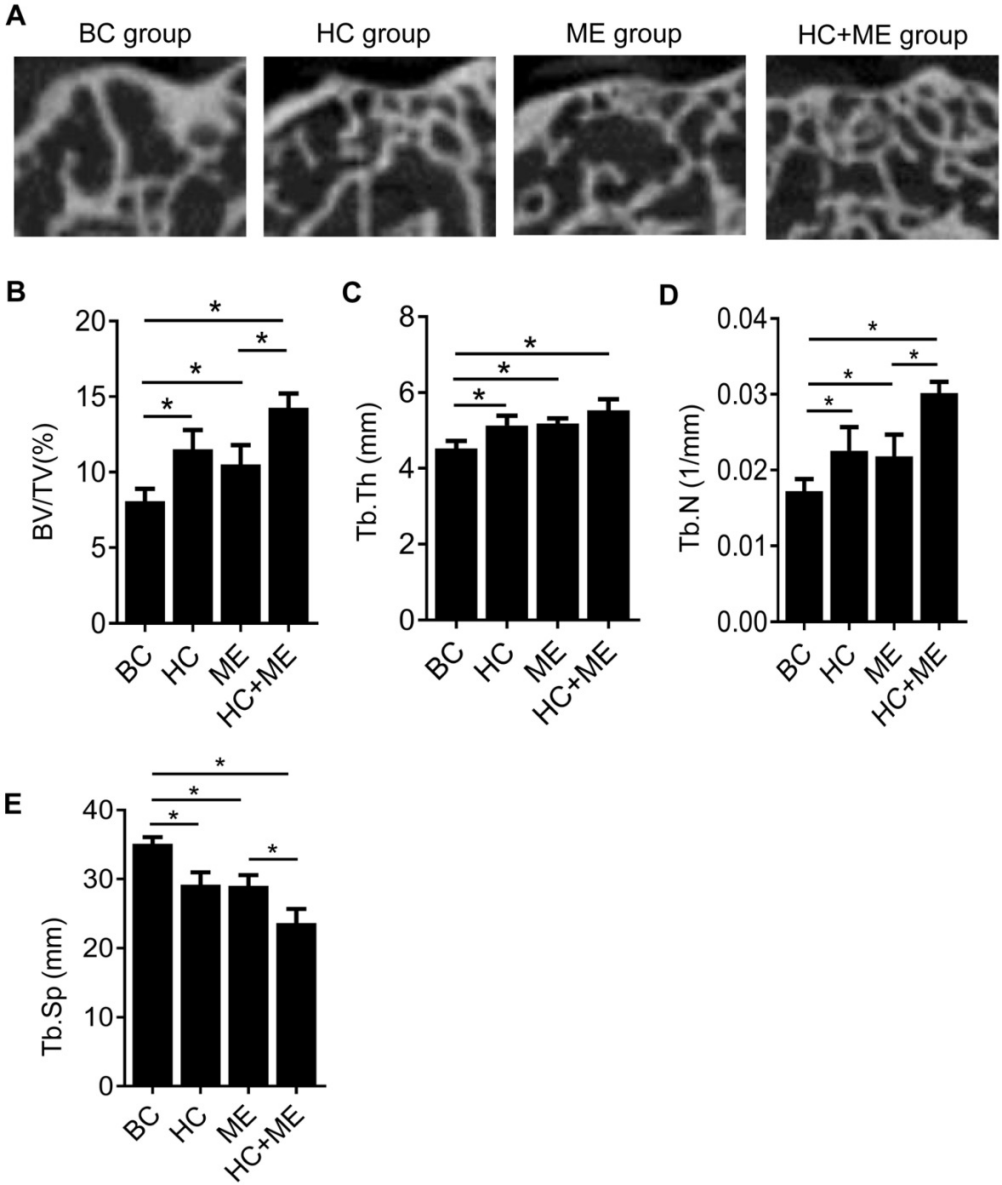
MicroCT analysis of the subchondral bone at the injury site and the correlation between articular cartilage thickness and relative bone volume (BV/TV). Representative micro-CT images of the distal femur in mice were shown (A). Quantitative analyses of trabecular bone volume fraction (BV/TV) (B), trabecular thickness (Tb.Th) (C), trabecular number (Tb.N) (D), and trabecular separation (Tb.Sp) (E). Data are represented as mean ± s.e.m. (*p< 0.05).

To determine whether HA-g-CS implantation and/or moderate-intensity exercise changed the subchondral bone formation by quantifying this process, we performed micro-CT analysis of the subchondral bone at the injury site in all groups (Figure 3A). Micro-CT analysis showed that HA-g-CS implantation or moderate-intensity exercise alone increased bone formation at the injury site compared to the no treatment group but combined treatments had a synergistic effect on bone formation as shown by further increased trabecular BV/TV (Figure 3B), Tb. Th (Figure 3C), and Tb. N (Figure 3D) and by decreased Tb. Sp as well (Figure 3E). We further performed correlation analysis in all the samples between articular cartilage thickness and BV/TV to evaluate the possible relationship between increased subchondral bone volume and the extent of cartilage repair.

### Characteristics of bone remodeling in the subchondral bone at the injury site in the mouse model

**Figure 4:**
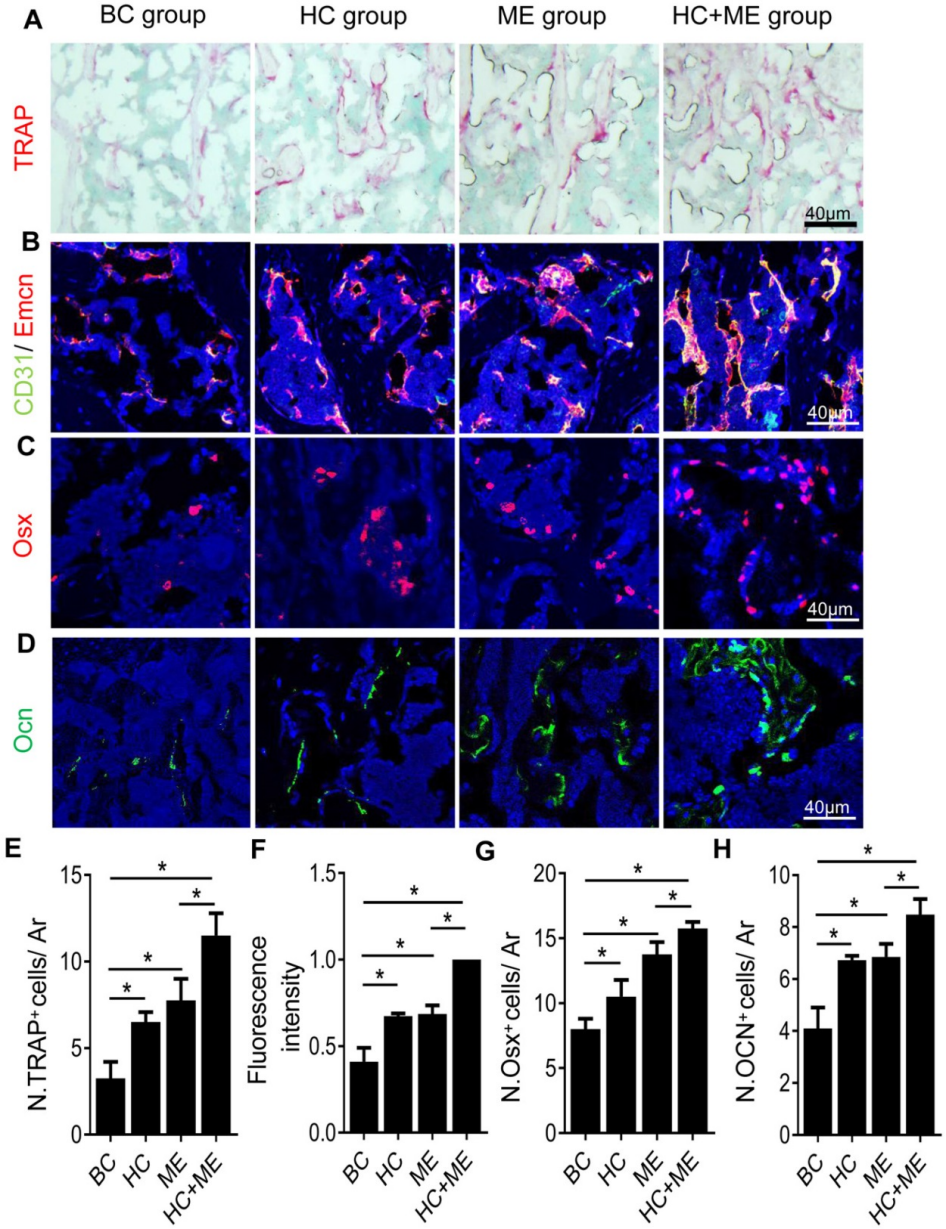
Characteristics of bone remodeling process in the subchondral bone at the injury site in the mouse model. Tartrate resistant acid phosphatase (TRAP) staining of the subchondral bone region at the injury site (A) and quantification of TRAP+ cells per mm^2^ tissue area (N. TRAP+ cells/ Ar) (E). Double immunofluorescence staining of CD31 (green) and Emcn (red) in subchondral bone sections were shown (B). Relative yellow fluorescence intensity showing double positive cells was measured (F). Immunofluorescence staining of osterix (Osx, red) (C) and quantification of Osx+ cells per mm^2^ tissue area (N. Osx+ cells/ Ar) (G). Immunofluorescence staining of osteocalcin (Ocn, green) (D) and quantification of Ocn + cells per mm^2^ tissue area (N. Ocn+ cells/ Ar) (H). DAPI stains nuclei blue. Data are represented as mean ± s.e.m. (*p< 0.05).

One possible explanation for the increased bone formation was that both HA-g-CS implantation and/or moderate-intensity exercise stimulated bone remodeling in the mouse model. Indeed, compared with BC group, HC, ME and HC+ME groups showed an increased number of TRAP^+^ mature osteoclasts in the subchondral bone at the injury site (Figures 4A, 4E), suggesting increased bone resorption. On the other hand, CD31^hi^Emcn^hi^ type-H vessels, a highly proliferative capillary believed to couple angiogenesis with osteogenesis 42, was also increased in the subchondral bone region at the injury site (Figures 4B, 4F). The numbers of Osx+ osteoprogenitors (Figures 4C, 4G) and Ocn+ mature osteoblasts (Figures 4D, 4H) were also increased in the same region, indicating that increased angiogenesis and osteoblastogenesis promoted bone formation in this area. Importantly, the combination regimen of HA-g-CS implantation and moderate-intensity exercise greatly promoted the subchondral bone remodeling as shown by a greater increase in the above parameters in HC+ME group compared with other groups.

### HA-g-CS implantation and/or moderate-intensity exercise promoted osteochondral repair in the mouse model by elevating both chondrogenesis and osteogenesis factors

**Figure 5:**
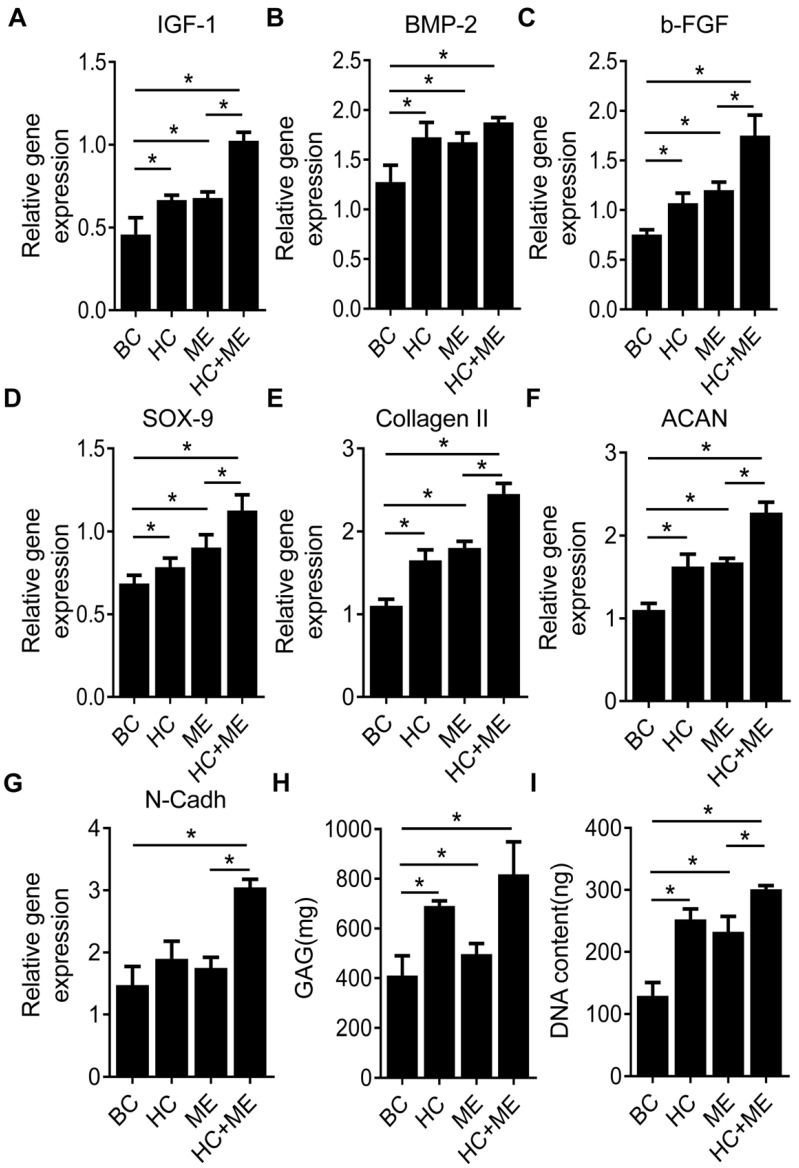
Characteristics of osteogenesis and chondrogenesis factors during osteochondral defect repair. Subchondral bone tissue from the four groups was harvested at 4 weeks after surgery and subjected to qRT-PCR to detect the mRNA levels of IGF-1 (A), BMP-2 (B) and b-FGF(C). Repaired cartilage tissue from the four groups was subjected to qRT-PCR to detect the mRNA levels of SOX-9 (D), Collagen II (E), ACAN(F) and N-Cadh(G). The glycosaminoglycan (GAG) content and the DNA content in the repaired cartilage tissue were measured (H and I). Data are represented as mean ± s.e.m. (*p< 0.05).

We compared the expression levels of typical growth factors in subchondral bone and repaired cartilage tissue. We found the expression levels of IGF-1, BMP-2 and b-FGF were increased in the subchondral bone region at the injury site in HC, ME and HC+ME groups compared with BC group (Figures 5A-5C). The expression levels of IGF-1 and b-FGF, but not of BMP-2, were significantly higher in HC+ME group than in other groups. We also examined the expression levels of typical chondrogenesis factors SOX9, collagen II, aggrecan (ACAN), and N-Cadherin (N-Cadh) in the repaired cartilage tissue (Figures 5D-5G). The expression levels of SOX9, collagen II and aggrecan were elevated in HC, ME and HC+ME groups compared with BC group, with the highest expression level in HC+ME group. And the expression level of N-Cadh was higher in HC+ME group than in other groups. Subsequently, we identified a higher DNA content and more GAG production in all treatment groups compared with BC group and they were significantly higher in HC+ME group than in other groups (Figures 5H-5I).

### HA-g-CS implantation and/or moderate-intensity exercise improve cartilage scores and relieve pain in the mouse model

**Figure 6:**
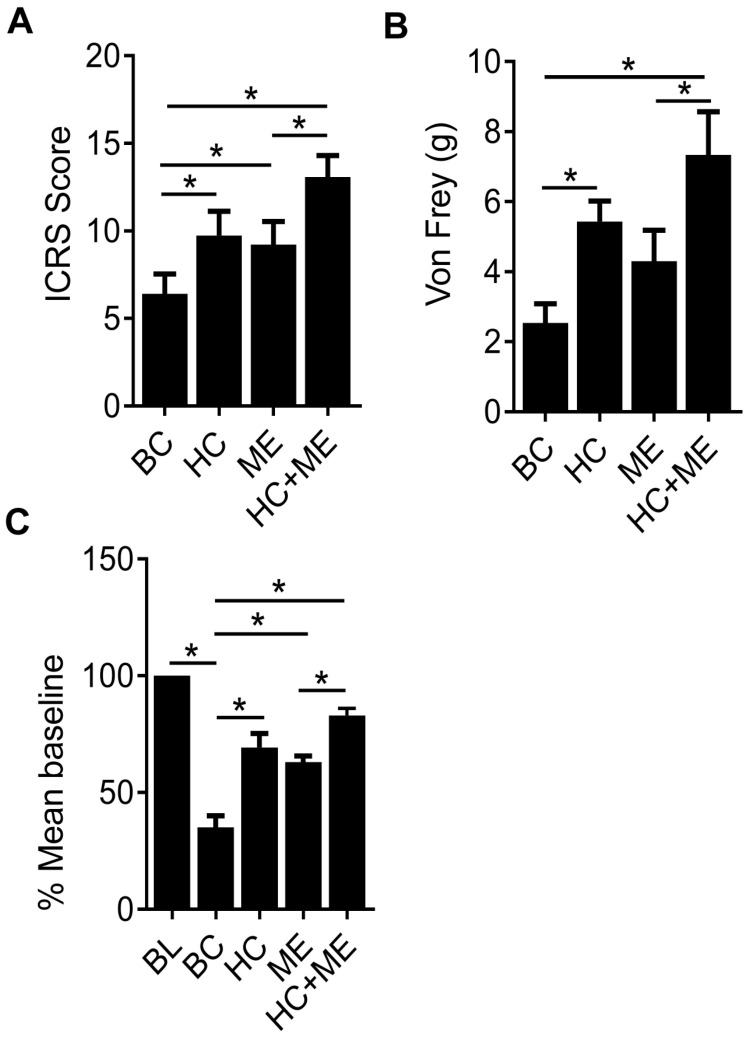
The International Cartilage Repair Society (ICRS) scores for repaired cartilage and the pain response of the mice. The ICRS scores for the four groups at 4 weeks after surgery were shown (A). Pain response of the mice in the four groups were measured by Von Frey test and running wheel experiment and the data were shown (B and C). BL, baseline. Data are represented as mean ± s.e.m. (*p< 0.05).

Finally, the International Cartilage Repair Society (ICRS) scores in the four groups (BC, HC, ME and HC+ME) were 6.66±1.47, 10.47±1.38, 9.23±2.07 and 12.94±1.67, respectively, showing that HA-g-CS implantation or moderate-intensity exercise alone significantly promoted cartilage regeneration in comparison with BC group at four weeks after surgery (P<0.05) (Figure 6A). And this effect was further intensified when the two treatments were applied in combination. Interestingly, we also observed a dulled pain response in all treatment groups as manifested by delayed response to physical stimuli (Figure 6B). Also, in the wheel running test, while the cartilage defect produced significant depression of running distance, mice in HC or ME group showed a steady increase in running at 4 weeks post-surgery, and the running distance in HC+ME group was restored to 83.75±1.73 percent of the baseline (Figure 6C).

## Discussion

Previous studies showed HA-g-CS implant 23, 26 or moderate-intensity exercise 27 alone promoted osteochondral regeneration. HA-g-CS provides the scaffold needed for tissue regeneration and hydroxyapatite supplement in the graft maintains a hydration state for the chitosan-hydrogel which further facilitates cell migration, differentiation and nutrient exchange 23, 26. Moderate-intensity exercise at designated time points of cartilage repair has been shown to provide mechanical stimulus beneficial for chondrogenesis of bone marrow stromal cells 29. In accordance with previous studies, our study showed that either of the two regimens promoted cartilage regeneration. However, we further unmasked a synergistic effect of the two regimens combined together on cartilage regeneration, as demonstrated by the better gross appearance and thickness of the joint cartilage, higher ICRS score and delayed pain response in HC+ME group. One of the most important reasons for unsatisfactory cartilage repair has been shown to be an insufficient number of bone mesenchymal stem cells (MSCs) migrating and remaining inside the healing site 43, 44. Increasing the number of MSCs by MSC-derived ECM scaffold or local injection of MSCs has been revealed to promote cartilage regeneration 45-48. Because we created, in our study, animal models of a full thickness osteochondral defect in which the articular osseous surface was also involved, the synergistic effect of HA-g-CS implant and moderate-intensity exercise combined together might have lain in increased mobilization and migration of MSCs into the subchondral bone at the injury site and further into the HA-g-CS implant at the cartilage defect.

Due to the avascular nature of the cartilage tissue, the metabolism and homeostasis of articular cartilage depends partly on its crosstalk with the underling subchondral bone 31, 32. It has been observed that, in normal conditions, products deriving from subchondral bone can be secreted into the joint cavity to reach articular chondrocytes 33. Also, signals deriving from subchondral bone regulates both the hypertrophy and survival of articular chondrocytes 35. The influence of subchondral bone on cartilage may be further amplified in a model of full thickness osteochondral defect because of removal of calcified cartilage in between and presence of micro-fractures on bone surface 32. Thus, in a model of osteochondral defect, repair of the articular surface relies on restoration of the whole bone-cartilage unit. Other studies have found that bone resorption and formation are increased in models of osteochondral defect 49. Our results demonstrated that both HA-g-CS implant and moderate-intensity exercise increased bone volume in the subchondral bone at the injury site. H-type vessels, characterized by high expression of endothelial markers CD31 and Emcn (CD31hiEmcnhi), promote perivascular osteoprogenitor cell survival by generating a unique microenvironment and link angiogenesis to osteoprogenitor cells 50. Many studies have also confirmed that subchondral bone neovascularity is characterized by the development of osteogenic coupled H vessels (CD31hiEmcnhi) 51-53.

The boosted bone formation was accompanied by increased type-H vessels and osteoclast-, osteoblast-lineage cells, suggesting an active bone remodeling activity. We also identified a positive relationship between cartilage thickness and bone volume. These results of ours indicate that subchondral bone and cartilage may recover in a parallel manner and either of the two regimens may have a beneficial effect on both cartilage and subchondral bone. Interestingly, we have found HA-g-CS implant combined with subsequent moderate-intensity exercise can further amplify the remodeling process and the final healing as well, compared with a single treatment.

Tissue regeneration requires growth signals to be produced in response to injury 54. In this study, we monitored changes in several key growth factors mediating bone and cartilage regeneration. We found both of our treatments promoted production of IGF-1, BMP-2 and b-FGF in the subchondral bone. Studies using IGF-1 null mice have shown that endogenous IGF-1 mainly affect mineralization process in bone 55, 57. However, in a rabbit model of osteochondral repair, *Zhang et al* 57 showed the effect of IGF-1 was dose dependent and injection of high-dose IGF-1 stimulated formation and integration of neo-cartilage while low-dose IGF-1 induced remodeling in subchondral bone. Importantly, they found low-dose IGF-1 induced expression of b-FGF rather than BMP-2 in the subchondral bone 57, suggesting that b-FGF and BMP-2, both important growth factors in bone formation, may be predominant on different phases 58-60. Study has shown that expression of b-FGF downregulated the level of BMP-2 and induced proliferation of osteoprogenitors while expression of BMP-2 dramatically promoted the mineralization rather than proliferation process 59. This discrepancy may be explained by the effect of hydroxyapatite in our study as previous studies have proved that hydroxyapatite has a potent capacity to both adsorb and stimulate the expression of BMP 61. We found that the expression levels of SOX9, type II collagen, aggrecan and N-Cadherin were up-regulated in cartilage. SOX9 is the key transcription factor for chondrogenesis which can induce the expression of cartilage extracellular proteins type II collagen and aggrecan 62, 63. As a result, contents of GAG and DNA are both increased. In the present study, we found HC+ME group had the highest expression of SOX-9 and other cartilage matrix protein genes, and the highest production of GAG and DNA, suggesting the best regeneration effect. Notably, IGF-1 and BMP-2 can both induce dramatic expression of SOX-9. Although we did not measure the levels of these two factors in repaired cartilage tissue, it is possible that the growth factors induced by HA-g-CS or moderate-intensity exercise in the subchondral bone might have promoted cartilage repair by enhancing the expression of chondrogenesis factors.

There are several limitations in our present study. First, we did not exclude the possibility that HA-g-CS or moderate-intensity exercise might act directly on the cartilage repair. Although we showed both the subchondral bone at the injury site and the repaired cartilage tissue produced more growth factors in treatment groups, it should be clarified by further experiments whether the growth factors produced specifically in bone formation might have induced cartilage repair. Moreover, the molecular mechanisms underlying the promotive effect of HA-g-CS or moderate-intensity exercise on cartilage repair and especially their synergistic effect are to be explored. Finally, our findings should be regarded as preliminary data due to our limited sample size and should be verified in more established animal models of osteochondral defect.

## Conclusions

This study has demonstrated that, in a mouse model of osteochondral repair, treatment of HA-g-CS or moderate-intensity exercise can improve the outcomes of cartilage repair. One potential mechanism is that either of the treatments may promote remodeling of the subchondral bone towards bone formation and thus cause release of osteogenesis as well as chondrogenesis factors. Combination of HA-g-CS implant and moderate-intensity exercise treatments can further intensify the promotive effect on cartilage repair. It is clinically potential that HA-g-CS implant plus moderate-intensity exercise may be a practical and promising therapeutic approach for repair of osteocartilage defects.
